# Qing-Xin-Jie-Yu Granules in addition to conventional treatment for patients with stable coronary artery disease (QUEST Trial): study protocol for a randomized controlled trial

**DOI:** 10.1186/s13063-016-1569-9

**Published:** 2016-09-15

**Authors:** Shengyao Li, Ming Guo, Huimin Mao, Zhuye Gao, Hao Xu, Dazhuo Shi

**Affiliations:** 1 Department of Cardiology, Xiyuan Hospital of China Academy of Chinese Medical Sciences, Beijing, 100091 China; 2 Graduate School, China Academy of Chinese Medical Sciences, Beijing, 100700 China; 3 Graduate School, Beijing University of Chinese Medicine, Beijing, 100029 China

**Keywords:** Stable coronary artery disease, Chinese herbal medicine, Qing-Xin-Jie-Yu Granules, Inflammation, Randomized controlled trial

## Abstract

**Background:**

Recurrent cardiovascular event remains high in stable coronary artery disease (SCAD), especially in patients with multiple risk factors, despite a high rate of use conventional treatment. Traditional Chinese Medicine (TCM) is a promising complementary and alternative medicine for treating SCAD, while evidence for its effect on long-term survival is limited. This study was designed to test if Chinese herbal medicine in addition to conventional treatment is more effective than conventional treatment alone in reducing major adverse cardiac event (MACE) for SCAD patients with multiple risk factors during a 1-year follow-up.

**Methods:**

This is a multicenter, placebo-controlled, double-blinded, randomized controlled clinical trial. A total of 1500 patients are randomized in a 1:1 ratio to receive the Qing-Xin-Jie-Yu Granules (QXJYG) or the placebo granules, twice daily for 6 months. The primary outcome is the combined outcomes including cardiac death, nonfatal myocardial infarction and revascularization. The secondary outcome is the combined outcomes including all-cause mortality, re-admission for acute coronary syndrome (ACS), heart failure, malignant supraventricular and ventricular arrhythmia influencing hemodynamics, ischemic stroke, and other thromboembolic events during 1-year follow-up. The assessment is performed at baseline (before randomization), 1, 3, 6, 9, and 12 months after randomization.

**Discussion:**

This is the first multicenter trial sponsored by the national funding of China to evaluate TCM in combination with conventional treatment on 1-year survival in high-risk SCAD patients. If successful, it will provide an evidence-based complementary therapeutic approach for reducing MACE from SCAD.

**Trial registration:**

The trial was registered in the Chinese Clinical Trial Registry on December 28, 2013. The registration number is ChiCTR-TRC-13004370.

**Electronic supplementary material:**

The online version of this article (doi:10.1186/s13063-016-1569-9) contains supplementary material, which is available to authorized users.

## Background

Over the past three decades, preventive and therapeutic agents have substantially improved the prognosis of stable coronary artery disease (SCAD), however, cardiovascular event remains high, especially in high-risk patients with additional multiple risk factors, such as hypertension, hyperlipidemia and diabetes mellitus, despite a high rate of use conventional treatment including aspirin, statins, β-blockers and renin-angiotensin-aldosterone system blockers, etc. [1]. In addition, increasing evidence supports a vital role of inflammation in the clinical instability of SCAD [2]. C-reactive protein (CRP), a systemic inflammatory marker, adds prognostic information to the cluster of traditional risk factors [3]. However, the novel anti-inflammatory drugs did not show a positive effect on further reducing major adverse cardiac event (MACE) [4, 5]. Therefore, it is necessary to seek more effective strategies for inhibiting inflammation and thus reducing the occurrence of MACE from SCAD.

Traditional Chinese Medicine (TCM) has been used to treat CAD for thousands of years. A meta-analysis of 70 randomized clinical trials including 9706 cases of CAD demonstrated a beneficial effect of TCM on clinical symptoms (odds ratio, 3.06 [95 % CI, 2.58 to 3.62]; *P* < 0.00001) and electrocardiograph (odds ratio, 2.46 [95 % CI, 2.16 to 2.80]; *P* < 0.00001) compared with isosorbide dinitrate, and no serious adverse effects were reported [6]. However, another systematic review pointed out that the majority of randomized controlled trials on CAD with TCM are of “poor quality”, thus more powerful evidence from high-quality clinical trials are needed for future clinical practice [7].

Qing-Xin-Jie-Yu Granules (QXJYG) have been used to treat CAD for nearly two decades in mainland China, and previous small-sample studies have demonstrated that QXJYG relieved angina pectoris and decreased cholesterol of patients with CAD [8]. QXJYG consist of five herbal granules, including *Astragalus membranaceus* (Huangqi), *Salvia miltiorrhiza Bunge* (Danshen), *Ligusticum chuanxiong Hort* (Chuanxiong), *Agastache rugosus* (Huoxiang), and *Coptis chinensis* (Huanglian). The pharmacological studies suggested that the bioactive component or structural modification of extracts from Huangqi [9], Danshen [10], and Chuanxiong [11] had effects on cardiac protection, plaque stabilization, inhibition of platelet aggregation, etc., and these three Chinese herbs are also widely used in TCM for treating CAD [12–14]. Moreover, extracts from Danshen and Huanglian exert anti-inflammatory effects [15, 16], and clinical studies showed that they decreased the level of high-sensitivity CRP (hs-CRP) in patients with cardiovascular disease [17, 18].

Therefore, QXJYG is worth investigating in high-risk SCAD patients due to the pleiotropic effect. The assumption is made that QXJYG in addition to conventional treatment may be superior to reduce inflammation of atherosclerosis and finally lead to the reduction of MACE in high-risk SCAD patients. If successful, it will provide an evidence-based complementary therapeutic approach for inhibiting inflammation and reducing MACE from SCAD.

## Methods/design

### Study objectives

**Fig. 1:**
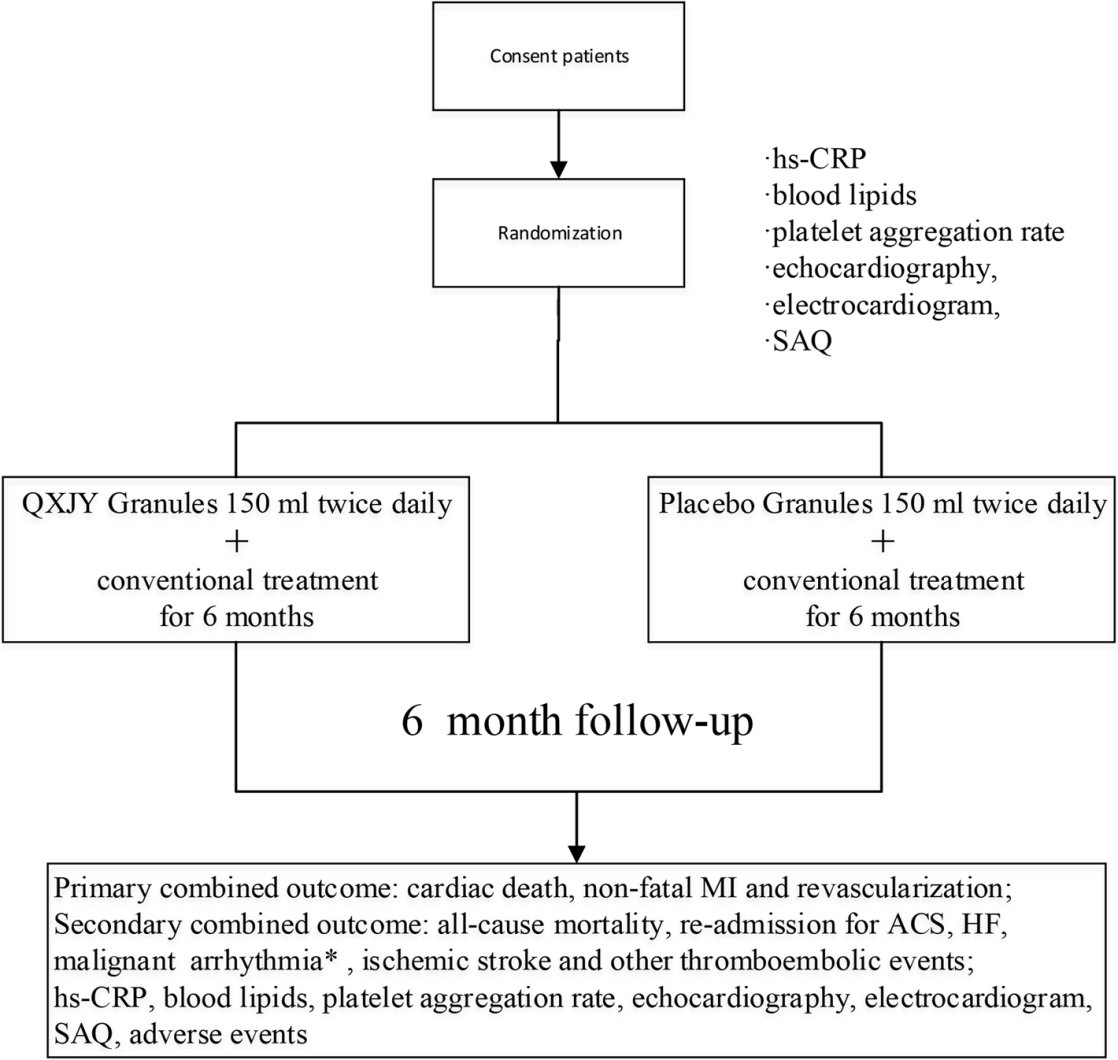
Study flow of the QUEST Trial

This study is designed as a multicenter, double-blind, randomized, placebo-controlled, parallel-group, superiority trial to test the hypothesis that QXJYG in addition to conventional treatment is more effective than conventional treatment alone in reducing MACE, which is defined as cardiac death, nonfatal myocardial infarction and revascularization, for SCAD patients with two or more atherogenic risk factors during 1-year follow-up (see Fig. 1).

### Design overview

The rigorous design, organization, and conduct of the trial are supervised by a Steering Committee, which comprises two members from each participating center in addition to the chairman, scientific coordinator and statistician. Eighteen clinical centers in mainland China will participate in the trial, including Xiyuan Hospital of China Academy of Chinese Medical Sciences, China-Japan Friendship Hospital, The First Hospital of Tianjin University of Traditional Chinese Medicine, etc. Data management and statistical analyses will be performed solely by data handlers and data analysts at Beijing Jiaotong University. This study follows the international recommendations for interventional trials [19]. (See the SPIRIT checklist in Additional file 1). All patients must personally sign and date an informed consent document before randomization.

### Inclusion criteria

The trial will recruit patients of both genders aged 18–75 years with evidence of CAD documented by previous myocardial infarction (>3 months before screening), percutaneous coronary revascularization (>1 month before screening), angiographic or cardiac computed tomography angiographic evidence of ≥50 % stenosis of ≥1 major coronary artery. Also required are two or more of the following four risks: a serum hs-CRP level ≥3 mg/L, a history of hypertension, hyperlipidemia, and diabetes mellitus.

### Exclusion criteria

The exclusion criteria are as follows:

1. Combined with congenital or rheumatic heart disease or severe heart failure;
2. Uncontrolled severe arrhythmia (including paroxysmal ventricular tachycardia and supraventricular arrhythmia), which can cause hemodynamic responses;
3. Acute cerebrovascular disease;
4. Uncontrolled blood pressure, with a systolic blood pressure ≥160 mmHg or a diastolic blood pressure ≥100 mmHg;
5. Severe primary hepatic, renal, hematologic or mental disorders;
6. Major head, chest or abdominal surgery within 4 weeks or bleeding tendency;
7. Pregnant or lactating women;
8. Suspected to be allergic to Chinese herbal medicine;
9. Currently participating in another clinical trial.

### Recruitment

Inpatients and outpatients with SCAD in each center will be screened. Each potentially eligible patient will be assessed by an attending physician for whether the patient should be recruited. The aim, procedures, and possible side effects of the study granules will be explained in detail to the patients; all patients will be asked to sign a written informed consent form before randomization. Neither financial nor nonfinancial incentives will be provided to attending physicians and patients for enrolment.

### Randomization and treatment assignment

Patients are randomized in a 1:1 ratio through a centrally controlled, computer-generated, site-stratified, block randomization schedule. The study granules are labeled with serial numbers, and each patient will be assigned the lowest number available at each participating center. All patients, care providers, attending physicians, laboratory staff and biostatisticians are blind to treatment assignment until the database has been locked. Randomization started on October, 2014. Enrolment of 1500 patients is expected to be completed in August 2016.

### Interventions

Eligible patients will be allocated to receive QXJYG or placebo for 6 successive months, in addition to conventional treatment including antiplatelet, lipid-lowing, antihypertensive or antidiabetic therapy, etc., according to the guidelines [20]. The QXJYG and placebo granules were produced and packed in a single batch (Production batch number:1405001H) by China Resources Sanjiu Medical and Pharmaceutical Co., Ltd., Shenzhen, China., which has no conflict of interest relevant to this study. The test results of drug quality were consistent with the Chinese Medicine Standards of the State Food and Drug Administration (SFDA). The placebo granules are composed of 10 % crude drug of QXJYG and 90 % starch, which have the identical appearance, smell, and scent as the active treatment granules. Patients will be instructed to dissolve the granules in 150 mL hot water and take orally twice daily for 6 successive months. Any other Chinese herbal decoction or Chinese patent medicine for treating CAD is prohibited during the study.

### Outcomes

The combined primary outcomes include cardiac death, nonfatal myocardial infarction, and revascularization. The combined secondary outcomes include all-cause mortality, re-admission for acute coronary syndrome (ACS), heart failure, malignant supraventricular and ventricular arrhythmia influencing hemodynamics, ischemic stroke, and other thromboembolic events during 1-year follow-up.

Hs-CRP, blood lipids, platelet aggregation rate, echocardiography, electrocardiogram, and Seattle Angina Questionnaire (SAQ) will also be monitored periodically.

### Safety assessment

Safety will be monitored up to 6 months after intervention and evaluated based on the incidence of adverse events (AEs) including clinically significant changes in physical examinations, vital signs, and standard clinical laboratory tests (complete blood count, blood biochemical examination, coagulation [including prothrombin time (PT), thrombin time (TT), international normalized ratio (INR), activated partial thromboplastin time (APTT), and fibrinogen] and urinalysis). The National Cancer Institute’s Common Terminology Criteria for Adverse Events (CTCAE) v4.0 grading system was used to classify the nature and severity of the AEs [21].

### Follow-up

The follow-up for all patients will be scheduled at 1, 3, 6, 9, and 12 months (see Additional file 2). The primary and secondary outcomes as well as safety will be evaluated at each follow-up. If severe adverse events (SAEs) occur, the attending physician must report to the principal investigator and ethics committee immediately, who will decide whether or not to continue the treatment. Patients are also free to continue or discontinue the study at any time during the trial. Additionally, any medication change and events leading to therapy interruption will be recorded on the case record form (CRF).

### Compliance

A mobile application will be used to remind the patients automatically at each follow-up appointment. In addition, each patient will be asked to offer at least two telephone numbers in order to be contacted in time. Furthermore, attending physicians will be responsible for each patient’s questions.

To assess medication adherence, patients will be asked to return the unused granules. Unused granules accounting for more than 20 % of the total granules will be defined as nonadherence. The reasons for nonadherence (e.g., discontinuation of intervention owing to harm or unsatisfactory efficacy) will be recorded on the CRF.

### Sample size

The sample size was calculated based on the expected reduction of incidence in MACE from pre- to post-treatment at 1 year. A previous study suggested that the MACE (death, myocardial infarction, and urgent revascularization) incidence in high-risk patients with SCAD is about 10 % within 1 year [1]. Assuming a MACE incidence reduction to 7 % following treatment with QXJYG added, and given a type I error rate of alpha = 0.05, a power of 80 % (type II error rate of beta = 0.2), the sample size for one arm needed to be 630, resulting in *n* = 2 × 630 = 1260 patients. Considering a dropout rate of 20 %, a total of 1512 patients needed to be allocated to reach the required number of patients for the efficacy analysis. For convenience of randomization, we decided to recruit 1500 patients.

### Statistical approach

Continuous variables will be presented as the mean ± standard deviation (SD), median or interquartile range (IQR), and as frequencies or percentages for categorical variables. The comparability of the characteristics between the two groups will be assessed using a two-sample Student’s *t* test for continuous variables and the chi-square test or Wilcoxon test, when appropriate, for categorical variables. For the SAQ scores, the analysis of covariance (ANCOVA) will be used to compare the change from baseline to 1 year between the QXJYG group and the placebo group [22]. Questionnaire score data, known as nonnormally distributed, will be transformed or analyzed with nonparametric ANCOVA. If appropriate, multivariable linear models will be used to assess the association between independent predictors and the continuous endpoints of the trial. Cumulative event rates of an outcome in the QXJYG and the placebo groups, respectively, will be estimated using the Kaplan-Meier method, and differences between the curves will be tested using the log-rank method. The crude and adjusted hazard ratio and their 95 % confidence intervals will be estimated by the Cox proportional hazards regression model.

The primary outcome will be conducted on the intention-to-treat (ITT) analysis. The ITT set includes all patients randomized to treatment. The per protocol set consists of all patients with no major deviation from the protocol and with an overall treatment adherence rate of 80 % or higher at the end of the study. The safety set consists of ITT patients excluding those who do not take any study medication or who have no record of follow-up after randomization. We will assess the effect that any missing data might have on the results by sensitivity analysis of augmented data sets. Dropouts will be included in the analysis by modern imputation methods for missing data.

We plan to conduct three subgroup analyses: first, we will compare hazard ratios of MACE based upon the level of hs-CRP (below 3 mg/L vs. greater than or equal to 3 mg/L). Second, we will compare the hazard ratios of MACE between SCAD patients with diabetes or not. Finally, we will compare the hazard ratios of MACE between men and women with SCAD.

For all analyses, a value of *P* < 0.05 is considered statistically significant, and all tests are two-tailed. *P* values will be reported to four decimal places. All analyses will be conducted using R software version 2.15.1 (http://www.R-project.org/), unless otherwise noted.

### Data management and monitoring

Data from all participating centers will be imported in the clinical data management system (CDMS) with the website at http://www.xyedc.com. Data and materials supporting the conclusions of this trial will be available in this system. A password system will be utilized to control access. Data integrity will be achieved through several mechanisms including referential data rules, valid values, range checks and consistency checks against data already stored in the database. Missing data or specific errors in the data will be detected by programs. Written documentation of changes will be available via electronic logs and audit trails. Original CRF will be kept at the participating center for 5 years after completion of the study.

### Safety monitoring

A Data and Safety Monitoring Board (DSMB) will be in charge of safety reviews, and will include at least two independent faculty members from the participating center. The safety will be evaluated for the first time when approximately 20 % patients have 6 months of data available. Subsequently, the DSMB will perform the interim analysis for safety with the support of the statistical group when approximately 50 % patients have 6 months of data available. In addition, the DSMB will conduct at least one on-site monitoring visit during the study to ensure that each participating center complies with the study protocol and Good Clinical Practice (GCP) principles; the data and SAEs are accurately and appropriately recorded on the CRF. When SAEs occur, the DSMB will perform unblinding immediately.

## Discussion

According to our knowledge, this is the first multicenter trial sponsored by the national funding of China to evaluate TCM in combination with conventional treatment on 1-year survival in high-risk SCAD patients.

Atherogenic risk factors including hypertension, hyperlipidemia, and diabetes are common in CAD patients, and clustering of them has a higher age-adjusted, long-term mortality risk [23]. In addition, accumulating evidence has revealed that all these traditional atherogenic risk factors are closely associated with an increased level of CRP, an inflammatory marker with additional prognostic value [24]. A study showed that in SCAD patients, the CRP concentration in the fifth quintile (>3.6 mg/L) showed an about twofold increase in MACE, compared with the first four quintiles [25]. This is in accordance with the consensus view that inflammation has a role in the pathogenesis of cardiovascular events. Therefore, inhibiting chronic inflammation has been regarded a potential strategy to reduce MACE from SCAD.

Chinese herbs have shown multifactorial cardiovascular protective effects, such as lowing plasma lipids [9], reducing oxidative stress [10], and inhibiting platelet aggregation [11], etc. In addition, the anti-inflammatory effects of Chinese herbs have gained much attention recently. Danshen and Huanglian, the two herbs used in QXJYG, also have been demonstrated in many animal or cell culture models to inhibit inflammatory respondence. In ApoE-deficient mice fed with a high cholesterol diet, salvianolic acid B, an extract from Danshen, reduced metalloproteinases-2 (MMP-2) and MMP-9 expression significantly [26]. In human umbilical vein endothelial cells as well as platelets, treatment with extract from Danshen attenuated tumor necrosis factor alpha (TNF-α)-induced expression of CD40. Furthermore, the expression of vascular cell adhesion molecule-1 (VCAM-1) and intercellular adhesion molecule-1 (ICAM-1) as well as the release of soluble VCAM-1, ICAM-1, interleukin-6 (IL-6), IL-8 and monocyte chemoattractant protein 1 (MCP-1) were downregulated [27]. Berberine, an extract from Huanglian, also decreased serum levels of TNF-α and IL-6 in rats with isoproterenol-induced acute myocardial ischemia [28]. This gives a stimulus for large controlled trials to examine the long-term effects of QXJYG in CAD patients, especially those in a high inflammatory state (a serum hs-CRP level ≥3 mg/L, with additional multiple atherogenic risk factors).

Granules have been widely used in mainland China. Our study QXJYG granules were produced strictly in compliance with standards of Good Manufactory Practice (GMP) and Chinese Pharmacopoeia 2010. The chemical compositions of the final products were analyzed for contamination with heavy metals, toxic elements, microbe and pesticide residues; the final products were analyzed for stability and adherence to quality standards of the CFDA. The fingerprint of QXJYG will be conducted to ensure the stability and homogeneity of the composition. The placebo granules have the identical appearance, smell, and scent as the active treatment granules. Granules were distributed to the 18 study sites with the same batch number. The company that provided the granules does not participate in the design, analysis, or interpretation of the study.

It should be noted that our study has limitations. First, the study is conducted only in mainland China, and whether the results from this trial can be applied to other ethnic groups is uncertain. However, the participating centers are located in different geographic areas in mainland China, and national minorities are also involved. Therefore, the recruited patients guarantee a certain representativeness. Second, the duration of follow-up is designed relatively short. The continuation of follow-up after the end of the study has been planned in order to assess the long-term outcome.

In conclusion, this trial is designed to demonstrate that the addition of QXJYG to conventional treatment will reduce the inflammation of atherosclerosis and finally lead to the reduction of MACE in high-risk SCAD patients.

### Trial status

The trial has currently recruited 1202 patients since October 2014, of which 906 have completed the 6-month follow-up. Thirty patients experienced MACE, but their allocated group is still bind. No SAEs have occurred to date.

## Additional files

Additional file 1: The SPIRIT Checklist. (DOC 123 kb) Additional file 2: SPIRIT figure. (DOCX 23 kb)

## Abbreviations

ACS: Acute coronary syndrome
AEs: Adverse events
APTT: Activated partial thromboplastin time
CAD: Coronary artery disease
CDMS: Clinical data management system
CRF: Case record form
CRP: C-reactive protein
DSMB: Data and Safety Monitoring Board
GCP: Good clinical practice
hs-CRP: High-sensitive C-reactive protein
ICAM-1: Intercellular adhesion molecule-1
IL-6: Interleukin-6
INR: International normalized ratio
ITT: Intention-to-treat
MACE: Major adverse cardiac event
MCP-1: Monocyte chemoattractant protein-1
MMP-2: Metalloproteinases-2
PT: Prothrombin time
QXJYG: Qing-Xin-Jie-Yu Granules
SAEs: Severe adverse events
SAQ: Seattle Angina Questionnaire
SCAD: Stable coronary artery disease
TCM: Traditional Chinese Medicine
TNF-α: Tumor necrosis factor alpha
TT: Thrombin time
VCAM-1: Vascular cell adhesion molecule-1
