# Supplementary material for: Qing-Xin-Jie-Yu Granules in addition to conventional treatment for patients with stable coronary artery disease (QUEST Trial): study protocol for a randomized controlled trial
Source: Trials. 2016 Sep 15;17:451. doi: 10.1186/s13063-016-1569-9 (PMC5024507; doi:10.1186/s13063-016-1569-9)
Supplement: Additional file 2: — SPIRIT figure. (DOCX 23 kb) [file 13063_2016_1569_MOESM2_ESM.docx]

|  | Baseline | Study | | | Follow-up | |
| --- | --- | --- | --- | --- | --- | --- |
| TIMEPOINT | 0 | 1Month | 3Month | 6Month | 9Month | 12Month |
| ENROLMENT: |  |  |  |  |  |  |
| Eligibility screen | X |  |  |  |  |  |
| Informed consent | X |  |  |  |  |  |
| Medical history | X |  |  |  |  |  |
| Physical exam | X |  |  |  |  |  |
| Allocation | X |  |  |  |  |  |
| INTERVENTIONS: |  |  |  |  |  |  |
| QXJYG |  |  |  |  |  |  |
| Placebo |  |  |  |  |  |  |
| Conventional treatment |  |  |  |  |  |  |
| ASSESSMENTS: |  |  |  |  |  |  |
| MACE ^a^ | X | X | X | X | X | X |
| Hs-CRP | X |  |  | X |  | X |
| Blood lipids | X |  |  | X |  | X |
| Platelet aggregation | X |  |  | X |  | X |
| Echocardiogram | X |  |  | X |  | X |
| Electrocardiogram | X |  |  | X |  | X |
| SAQ | X | X |  | X |  | X |
| Coagulation function^b^ | X |  | X | X |  |  |
| Liver and renal function^c^ | X |  | X | X |  |  |
| Routine tests^d^ | X |  | X | X |  |  |
| Adverse events | X | X | X | X | X | X |
| Concomitant medications | X | X | X | X | X | X |
| Compliance |  | X | X | X | X | X |
